# From *Hydra* to Humans: Head Activator in Neurogenesis and Neurorepair

**DOI:** 10.3390/cells15070616

**Published:** 2026-03-30

**Authors:** Andrii Klymenko, David Lutz

**Affiliations:** 1 Department of Neuroanatomy and Molecular Brain Research, Ruhr University Bochum, 44801 Bochum, Germany; 2 Department of Anatomy and Cell Biology, Medical University—Varna, 9002 Varna, Bulgaria; 3 Vascular Biology Research Group (RenEVA), Research Institute, Medical University—Varna, 9002 Varna, Bulgaria

**Keywords:** regeneration, neurorepair, neurodegeneration, small-molecule therapeutics

## Abstract

*Hydra vulgaris*, an ancient cnidarian, exhibits remarkable regenerative and neurogenic abilities, mediated by morphogenetic peptides, particularly the head activator peptide. This neuropeptide appears to regulate cell proliferation, differentiation, and nerve net maintenance in hydra and, surprisingly, exerts similar mitogenic and neurogenic effects in mammalian systems. Despite early enthusiasm, research on head activator has declined, due to controversies about its genetic origin, receptor identity, and artefacts generated during isolation. Nonetheless, a synthetic variant of head activator corresponding to the described sequence has consistently exhibited strong biological activity in a variety of mammalian cells. Experimental evidence implicates the sortilin-related receptor (SorLA) as a primary receptor in mammals, with potential modulatory roles for the G-protein-coupled receptor GPR37. This review consolidates current knowledge on the evolutionary context, molecular characteristics, and functional activities of head activator. Insights from mammalian systems highlight its pleiotropic effects across species. Given its neuroprotective, regenerative, and immunomodulatory properties, head activator may merit reconsideration as a therapeutic candidate for neurodegenerative disorders and regenerative medicine.

## 1. Introduction

Some of the earliest organisms to develop body symmetry and a simple neuronal net belong to the ancient clade *Cnidaria*, which branched away from the bilaterians 850–635 million years ago [1]. One representative, *Hydra vulgaris* or simply hydra, has been studied as a classical model organism for morphogenesis, neurogenesis, and regeneration [2,3]. Hydra has a nearly unlimited regenerative and stem cell renewal capacity [4]. At the same time, hydra possesses one of the first primitive forms of a neuronal net to evolve on Earth; it is strikingly intricate in its complexity [3].

**Figure 1:**
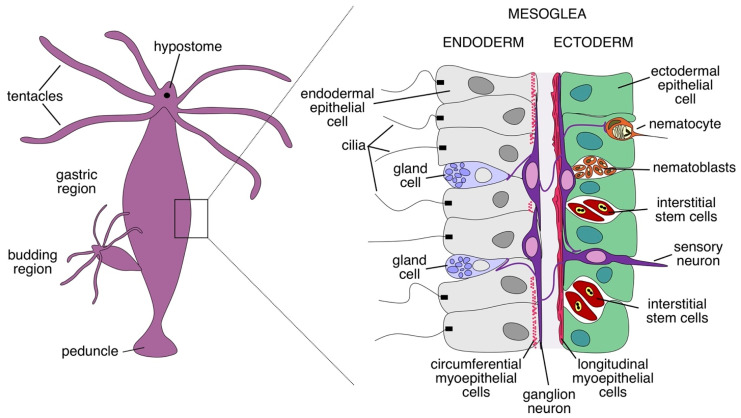
Anatomy of the hydra. The body column of the hydra is composed of a peduncle (foot region), gastric region, and head region, with an oral opening called the hypostome, surrounded by tentacles. The reproduction of hydra occurs primarily asexually through budding, resulting in genetically identical clones. The body consists of the endoderm (gastroderm), mesoglea, and ectoderm. The main cell types are interstitial, epithelial, and neuronal. A pool of pluripotent interstitial cells gives rise to all cell lineages. The epithelial cells can differentiate into secretory gland cells or into armed nematocytes, while ganglion and sensory neurons form the gastrodermal and ectodermal nerve nets. Adapted from [5].

The hydra is a small predatory fresh-water polyp that reproduces mostly asexually. It is primitively organised, with a tubular body subdivided into the basal disc (foot), body column (gastric region), and head (Figure 1). The body can reach up to 30 mm in length. A hypostome with an oral opening, surrounded by a radially symmetrical crown of tentacles forming the head region. It is settled on top of the body column with the gastric cavity, where the microbiome resides and prey digestion occurs. The body is composed of two layers: the outer epidermis, and the inner gastrodermis, separated by mesoglea, a layer of extracellular matrix. Three main cell types shape the histological architecture of hydra: epithelial cells, interstitial cells, and neurons (Figure 1).

The epithelial cells outline the epidermal and gastrodermal layers. These cells have epitheliomuscular features; hence, they are alternatively denominated as myoepithelial cells. They follow a circular arrangement in the gastroderm, and a circular one in the ectoderm, providing body movements [6]. These cells can proliferate upon injury and differentiate into more specialised entities called “cnidocytes”, harbouring stinging organelles (nematocysts) [7]. The cnidocytes densely populate the tentacle areas of hydra, being enriched in neurotoxic substances, ready for a rapid injection to paralyse different kinds of prey. Captured by the tentacles, the immobilised prey is propelled towards the hypostome. Alternatively, the epithelial cells outlining the endoderm can transfigure into secretory gland cells to release mucus and digestive enzymes [6].

**Figure 2:**
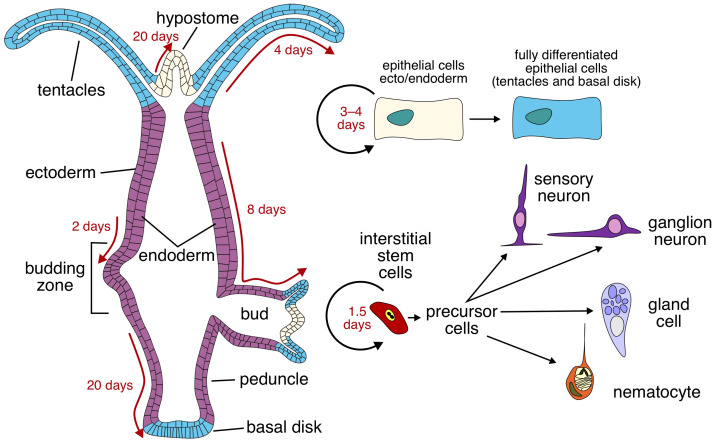
Regeneration and differentiation cycles in hydra. The hydra resides in a perpetual state of cell renewal driven by a pool of pluripotent interstitial cells. These cells can give rise to all lineages: epithelial, neuronal, and interstitial cells. Proliferating and differentiating cells continuously replenish the endodermal and ectodermal layers, allowing for regeneration and budding. Adapted from [5].

The interstitial cells of hydra represent an equivalent of stem cells with a high mitosis turnover to maintain a pool of pluripotent cells and replenish epithelia or neurons through terminal differentiation (Figure 2). This allows for a full recovery from even the most severe injuries such as decapitation or a complete disaggregation into a suspension of cells. Driven by its interstitial cells into a continuous state of cell renewal and adolescence, the hydra escapes senescence to maintain biological longevity [8]. Strikingly, some of the molecular mechanisms underlying longevity in hydra are shared by vertebrates and, specifically, mammals. In particular, the telomeric sequences are similar in cnidarians and mammals [9,10]; orthologous aging-related genes, such as FoxO, myc, and PIWI, were found in both cnidarians and mammals [8].

In simple terms, the neurons of hydra build two major functional groups: sensory neurons and ganglion cells. Deeper molecular analyses have revealed a highly complex architecture encompassing at least 11 neuronal subtypes with specific biomarkers, localisation, and unique developmental trajectories [11]. For instance, Hym-176 positive neurons single-handedly form the motor network [12]. These cells build a diffuse nerve net scattered across the entire body of the hydra. In fact, there are two separate nerve nets without apparent synaptic connections between each other—one in the epidermal layer and another in the gastrodermal layer [13,14]. In addition, there are hypostomal and peduncle nerve ring regions, which are densely populated by neurons [15]. It should be emphasised that, due to the common origin of bilaterian and cnidarian nervous systems, a high number of key neurogenesis-regulator genes have shared orthologs, e.g. CREB, POU, LIM, Sox/TCF, Notch, Wnt, Hedgehog, and JAK/STAT [16].

The epithelial and neuronal cells secrete a wide range of signalling molecules, most of which are peptides [17]. These peptides form a rich neuroendocrine system, covering every aspect of the hydra’s life—they control development and regeneration, neurotransmission, behavioural responses, and, especially, pathogen defence. Previous research efforts have been mainly made to study antibacterial peptides, cytotoxic compounds for cancer treatment, or the use of cnidarian neurotoxins to modulate ion channels in the context of neurological disorders such as neuropathic pain and epilepsy [18,19]. One short peptide of hydra, called “head activator”, has been shown to influence neural differentiation and regeneration in hydra. Furthermore, synthetic head activator has induced mitogenic and neurogenic responses in mammalian tissues and cell lines [20]. Given the substantial evolutionary divergence between cnidarians and vertebrates, however, potential parallels in functions as well as evolutionary conservation amongst species should be interpreted cautiously.

Between 1970 and 1990, pioneering studies on head activator peptide provided fundamental insights into neurogenesis and neuropathology in both cnidarians and mammals, albeit becoming a subject of considerable debate within the scientific community [21] due to conflicting findings and unresolved questions, particularly concerning its gene of origin and aspects of its signalling pathway.

As the gene sequence coding for head activator has not been conclusively identified, synthetic versions of the peptide have consistently displayed mitogenic and regenerative activity in preclinical mammalian models. These observations support the functional relevance of head activator as a regenerative therapeutic despite the uncertainty about its endogenous origin. In this review, we synthesise the existing knowledge of the roles of head activators in regeneration and neuroprotection and provide a link to human neuropathology and potential pharmaceutical uses.

The abundance of experimental evidence has sparked interest in cnidarian peptides as possible stimulants of regeneration and as neuroprotectants.

## 2. Peptidergic Signalling in Hydra

Hicklin and colleagues have identified head and foot organisers in hydra as central hubs of development and maintenance of the body and neuronal net, mediated through intrinsically secreted morphogenic factors [22]. The pioneering works of Chica Schaller, Toshio Takahashi, Toshitaka Fujisawa, and their colleagues have discovered the underlying signalling peptides of morphogenesis in hydra [23,24,25]. The predominantly peptidergic signalling in hydra consists of a wide palette of peptides with different and sometimes antagonistic functions [25]. These peptides are classified into epitheliopeptides [26] and neuropeptides [27] according to their cellular origin, and further subdivided with respect to their structural and functional hallmarks.

In Schaller’s laboratory, chromatographic fractions of hydra homogenates have displayed morphogenic activity; further separation techniques led to the isolation of head activator neuropeptide [28] and foot formation factors such as the epithelia-peptides pedin and pedibin [29]. These peptides have been shown to control axial body patterning by shaping the head and foot regions in hydra as well as to regulate stem cell proliferation and neuronal differentiation [29,30].

Subsequent large-scale analysis within the scope of The Hydra Peptide Project identified nearly 800 peptides, out of which only 55 were functionally characterised [25]. Different peptide families have been discovered: morphogenic, neurogenic, myoactive, and behavioural regulators [25]. We have summarised some morphogenic and neurogenic peptides in Table 1.

**Figure 3:**
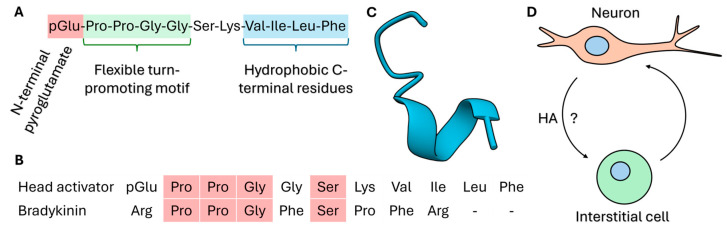
Amino acid sequence, structure, and proposed biological activity of hydra’s head activator: (**A**) amino acid sequence of head activator highlighting key residues in different colours; (**B**) similarities in residues within the amino acid sequences of head activator and bradykinin highlighted in colour; (**C**) predicted conformation of head activator based on the residues in the amino acid sequence using AlphaFold 3 [38]; and (**D**) head activator (HA) in hydra is predominantly understood to stimulate interstitial cell proliferation and neuronal differentiation.

The main morphogenic peptides in hydra can be classified into head- and foot-specific factors. Some peptides have a dual function and participate both in morphogenesis and neurogenesis. The head-specific peptides include head activator and HEADY. Head activator is an undecapeptide with the sequence pGlu-Pro-Pro-Gly-Gly-Ser-Lys-Val-Ile-Leu-Phe and a molecular mass of approximately 1.2 kDa (Figure 3). The peptide contains an N-terminal pyroglutamyl residue (pGlu), which arises from the cyclisation of glutamine and represents a common modification in neuropeptides that increases resistance to proteolytic degradation. Interestingly, head activator shares partial similarity with bradykinin; however, bradykinin does not reproduce the neurotrophic effects attributed to head activator [34]. Earlier spectroscopic data suggested a β-sheet dimer structure of the peptide [35,36]; however, subsequent biophysical analyses have shown that the peptide is largely monomeric and does not adopt a stable secondary structure in solution [37]. Although the existence of an endogenous head activator in hydra remains debated, the peptide has been reported to induce development of the head region and to promote mitotic expansion of the interstitial cells, followed by enhanced differentiation into epithelial and neuronal cells [30].

Another morphogenic entity, the foot-formation factor, was identified as containing the pedin and pedibin peptides [29]. Both peptides originate from epithelial cells and have a similar function: they induce development and regeneration of the foot region [29]. Additionally, pedibin participates in head and tentacle organisation [39], while pedin can support axial body patterning and asexual reproduction by budding [40].

**Figure 4:**
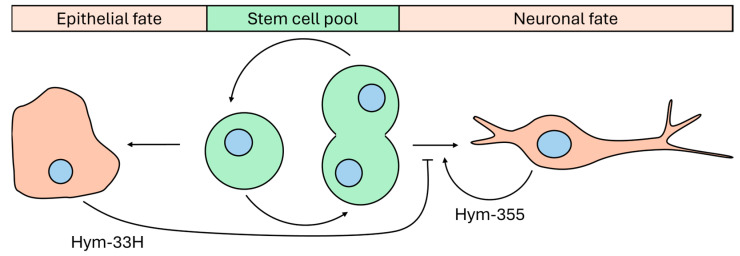
Regulation of neurogenesis and morphogenesis through interactions between small signalling peptides in hydra. Interstitial cells maintain a pool of pluripotent progenitors that can differentiate into epithelial or neuronal lineages. Fully differentiated cells secrete bioactive peptides that can regulate cellular homeostasis. Neuronal cells produce Hym-355, which promotes neuronal differentiation and supports maintenance of the nerve net. In contrast, epithelial cells release Hym-33H, which functionally antagonises Hym-355 and inhibits neuronal expansion. Only Hym-355 and Hym-33H are shown here as representative examples.

The maintenance and formation of neural networks in hydra is controlled by stimulatory peptides, such as pedin, head activator, and Hym-355 [27,41], and also by inhibitory peptides such as Hym-33H, Hym-35, Hym-37, and Hym-310 [27,41]. The stimulatory peptides induce proliferation of the interstitial stem-like cells and their differentiation into a neuronal phenotype, while the inhibitory peptides strive to keep a conserved state by showing an inverse effect [25]. The interaction between these two systems is an equilibrium, which can, however, adaptively be shifted, e.g., upon injury. In particular, the inhibitory Hym-33H and the stimulatory Hym-355 are antagonistic peptides that form a regulatory feedback loop depending on the density of the secreting cells and corresponding relative peptide concentrations (Figure 4), thus enabling the tight surveillance of neuronal differentiation [32,42].

The most prominent stimulatory peptide is Hym-355, while other neuronal stimulators, such as head activator and pedin, likely modify the neuronal differentiation response. Hym-355 is a neuropeptide expressed in the head and foot region and endodermal nerve net [32]; notably, it contains a C-terminal proline-arginine-glycine-amide (PRGamide) motif that corresponds to mammalian Arg-vasopressin [43]. The epitheliopeptide pedin is present in both the endoderm and ectoderm. Having a dual mitogen and morphogen function, it participates in axial body patterning and stimulates proliferation and neuronal differentiation [29]. Of note, pedin is related to an actin-binding protein, thymosin-β_4_ [40]. Head activator peptide exerts a dualistic functional nature as a mitogen and neuromorphogen, and is predominantly expressed in the ectodermal neurons [44].

The peptides inhibiting neuronal differentiation belong to the proline–tryptophan family (PW family) and contain an eponymous C-terminal proline–tryptophan residue [25]. An example of these peptides is Hym-33H, which is mainly expressed in the ectodermal epithelium of the body column and understood to inhibit neuronal differentiation across the entire nerve net by antagonising Hym-355 [42]. However, prolonged exposure to Hym-33H neutralises this inhibitory effect and suggests, therefore, an autoregulatory loop [42].

Such a complex interplay between the peptides allows for targeted control of morphogenesis, neurogenesis, and regeneration. We hypothesise that cnidarian peptides, including head activator, may potentially exert comparable neuromorphogenic effects in mammalian tissues, analogous to those observed in hydra.

## 3. Context-Dependent Signalling of Head Activator

Although the existence of an intrinsic head activator in vivo remains debated, synthetic peptide variants have consistently displayed mitogenic and neurogenic activity. Following this notion, we will first discuss the discovered effects of the peptide to provide a basic understanding of its functions; later in this review, we will refer to the in vivo existence of the peptide. 

**Figure 5:**
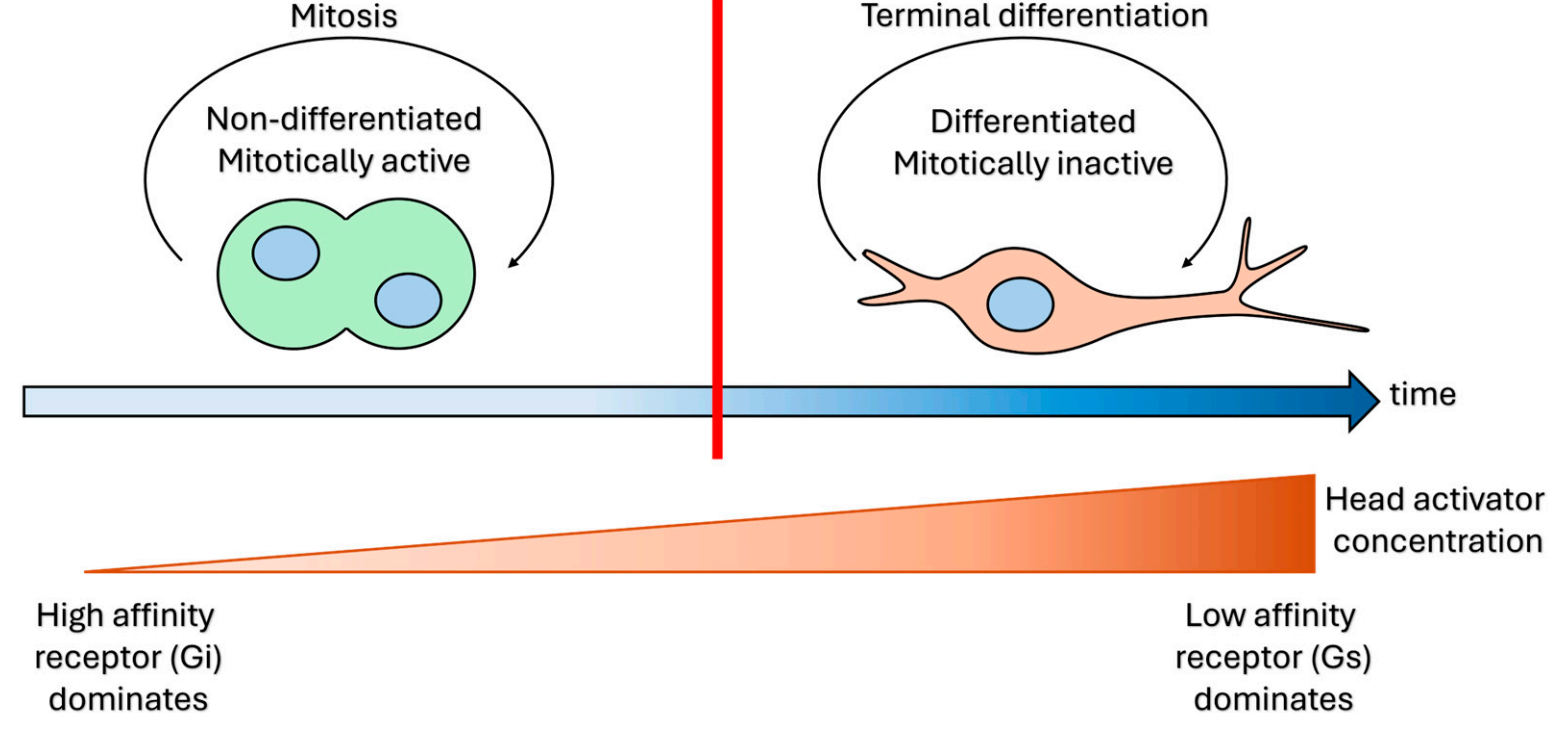
Determinants of the context-dependent mode of action of head activator peptide. The effects of head activator on the cells are temporally dynamic and depend on the differentiation status and mitotic activity of the cells. The main difference between immature and mature cells lies within the expressed head activator receptors. The immature cells express the high-affinity receptor, which requires a minimal concentration of the head activator peptide to become activated. It is a Gi-coupled receptor, which inhibits the adenylyl cyclase and depletes the concentration of cAMP; this signalling cue induces proliferation of the cells. In maturating cells that lose the ability to divide, a low-affinity Gs-coupled receptor dominates, which induces cAMP upregulation to assist in the differentiation process.

In hydra, head activator has been reported to regulate formation of the head region, cell division, and neuronal differentiation [30,45,46,47,48]. The molecule has been postulated to act as a growth factor for early development of the nervous and neuroendocrine systems in mammals [20]. Head activator seems to have two temporally dynamic and context-dependent modes of action controlled by different pathways in hydra (Table 2 and Figure 5). As an early ontogenetic cue, the molecule stimulates proliferation of mitotically active cells [49,50]. As a later ontogenetically occurring signal in post-mitotic cells, it sets the course of terminal differentiation into neurons or head-specific epithelial cells to form the nerve net and head organiser [49,50]. It has been shown that the border between differentiation and proliferation signals lies within the concentration of head activator: under a low dosage (10^−13^ M), the interstitial and epithelial cells begin to proliferate, whereas when the concentration rises up to 10^−11^–10^−9^ M, the cells initiate head-specific epithelial and neuronal differentiation [47,51]. Several kinetic studies have determined that hydra possesses at least two types of head activator receptors, one with a high affinity, which is responsible for the mitogenic effect, and another with a lower affinity, which triggers cell differentiation [52,53,54], thus explaining the dose–effect relationship through different receptor affinities. Binding studies indicate the preferential attachment of head activator to the surface of immature cells over fully differentiated cells, thus favouring cell cycle phase and differentiation status-dependent receptor expression patterns [55]. Moreover, an autocrine growth factor function of head activator has been proposed to control the G2/S transition during mitosis [55]. The early pro-mitotic activation inhibits the adenylyl cyclase and depletes cAMP, while later-occurring neuronal differentiation requires cAMP upregulation [50,51,56]. Of note, similar temporal fluctuation patterns of cAMP concentration after head activator treatment were observed in rodent livers [57]. The radically opposing effects of head activator on cAMP concentration indicate that both a stimulatory and an inhibitory G-protein-coupled receptor (GPCR) are likely involved. However, if both receptors were simultaneously active, they could possibly cancel each other out. We assume, therefore, that their activity or expression must be controlled in a temporally dynamic manner, enabling one single receptor class to dominate during the corresponding phase of cell cycle or differentiation, as Schaller and colleagues have previously shown [55].

## 4. Signalling Pathways of Head Activator Peptide in Hydra

Upon secretion from the neurons, if this occurs, head activator can become inhibited through dimerisation and proteolytic degradation [58], or can bind to a carrier molecule to escape proteolysis and preserve its activity [59]. However, an NMR-spectroscopic analysis has questioned dimerisation as an inactivation mechanism [37]. Furthermore, the dimer has also been reported to be a potent mitogen [60]. The carrier molecule of head activator peptide, head activator-binding protein (HAB), has been identified as a high-affinity receptor that exists in both membrane-anchored and soluble forms [60,61]. Interestingly, HAB is related to the mammalian sortilin-related receptor, SorLA, also known as SORL1 [61,62], a multifunctional intracellular trafficking protein located in the Golgi apparatus, which can interact with multiple ligands such as apolipoprotein E, amyloid precursor protein, and neurotrophic factors [63]. Furthermore, the mammalian homolog SorLA has been shown to interact with head activator and facilitate its signalling [64]. HAB/SorLA seems to be the central entity and very first step in the head activator signalling cascade. We believe that this protein could context-dependently engage in a complex with either a Gs or a Gi protein, considering the downstream regulation of the cAMP as a second messenger [56]. Indeed, the existence of such a complex seems convenient, since SorLA alone would not be sufficient to trigger cAMP concentration changes due to SorLA lacking any association with G proteins. Rezgaoui and colleagues proposed a receptor complex consisting of SorLA and inhibitory GPR37 to facilitate mitotic signalling in mammalian cells [65]. This could indicate that a similar receptor complex may be present in hydra. However, there are no known GPR37 orthologs in hydra, leaving the identity of the cnidarian head activator mitotic co-receptor obscure. Another supporting thesis about a receptor complex was reported by Jacobsen and colleagues [64]. They showed that monomeric SorLA binds head activator with a lower affinity in a purified SorLA/head activator system [64]. Earlier, Hampe and colleagues observed a high-affinity binding between the dimerised head activator and SorLA in a pull-down experiment with NT2 cell lysates [60]. The authors showed that this discrepancy cannot be attributed to the different monomer and dimer affinities, since both conformations are nearly equipotent [64]. They suggested that SorLA requires a partner protein, such as a co-receptor, to reach a high-affinity interaction with head activator [64]. In a pull-down fraction, a wide range of proteins is present, including the putative parter of SorLA, leading to high-affinity binding, while in a purified system with only SorLA present, the receptor partner must be missing, resulting in a low affinity.

Although the mechanism of controlling neuronal differentiation by head activator has remained enigmatic, there are some data indicating the involvement of a stimulatory GPCR. It appears very likely that this pathway induces activation of the adenylyl cyclase (AC) and adenylyl cyclase associated-protein (CAP), causing the intracellular concentration of the cyclic adenosine monophosphate (cAMP) to rise [56]. Of note, the CAP proteins can regulate actin filament dynamics [66] and possess a high degree of homology between cnidarians and mammals [56]. Upregulation of cAMP results in activation of the cAMP response element-binding protein (CREB) transcription factor, likely through the protein kinase A (PKA); the activated CREB then modulates the transcriptional activity of differentiation-associated target genes [51]. Of note, CREB is evolutionarily conserved between hydra and mammals [51]. Synthesis of the presented data implies that head activator signalling cascade seems to be heavily dependent on the regulation of the AC-cAMP-PKA axis, which is, indeed, one of the central entities in regenerative processes across multiple species and cell types. This pathway has been reported to be involved in head regeneration [67], neuronal determination, and differentiation [51] in hydra. HAB or SorLA is likely the primary receptor that facilitates head activator signalling; however, the resulting effect depends on the previously described dynamic conditions and differential co-receptor expression. The identity of head activator G-protein coupled co-receptors that could regulate this pathway remains unknown.

## 5. Effects of Synthetic Head Activator Applied to Mammals

Strikingly, the mitogenic and neurogenic responses observed in hydra could be reproduced in mammals through the application of head activator both in cell cultures and in vivo. Mammalian cell culture experiments showed that the addition of head activator to the culturing medium elevated mitosis rates of different cell lines, in particular, the P19 embryonal carcinoma cells [68], NH-15-CA2 neuroblastoma-glioma cells [44,69], NT2 neuronal precursor cell line [60], BON neuroendocrine cell line [60,70], COS-7, HEK-293 [65], and AtT20 cells [30].

Furthermore, the exogenous application of head activator peptide induced neuronal differentiation and stimulated neurite formation in murine neuroblastoma Neuro-2A cells [34] and in P19 embryonal carcinoma cells [68]. Supplemented head activator promoted the survival of dorsal root ganglion neurons and paravertebral sympathetic ganglion neurons isolated from chicken embryos in a culture devoid of neurotrophic factors that would have otherwise not remained viable for a long time [34]. Similarly, head activator stimulated the growth of cultured embryonal chicken brain cells [71]. The neurotrophic activity in culture, and the observed cerebral expression of head activator in vivo, led Schaller and Bodenmüller to conclude that head activator may have a similar function in the human nervous system as that observed in hydra, acting as a regulator of neural development [20].

A range of experiments with the in vivo application of head activator in rodents and cats showed its rapid systemic distribution [72] and differential tissue- and dose-dependent pleiotropic effects across multiple organs (Table 3).

Head activator is hydrophobic, with an estimated half-life of less than 30 seconds; after administration, the peptide is quickly absorbed by various organs and transformed into hydrophilic derivatives [72]. The effects of head activator could be observed at dosages of 10–100 μg/kg; furthermore, some effects were dose-dependent, and the responses varied based on the administered dosage (Table 3), favouring different context-dependent signalling mechanisms akin to cnidarian signalling.

Notably, the application of head activator to pregnant rats elevated the number of full-term pregnancies from 66.3% in the control to 100% in the treated animal groups [80]. Sakura and colleagues detected head activator in the human placenta, specifically in the trophoblasts, and in the maternal bloodstream. Additionally, head activator’s concentration was shown to increase over the course of gestation [88]. This suggests that head activator may support the placenta in maintaining foetal development. We assume that this effect could be used to counteract placenta failure and prolong the intrauterine period sufficiently for foetus maturation.

Head activator has been shown to stimulate epithelial proliferation in the tongue, trachea, cornea, and pylorus, but was not shown to affect the smooth myocytes in the respiratory and gastrointestinal tract [73,74,76]. These results imply that head activator could stimulate wound epithelialisation both for surface wounds and mucous membrane lesions. Additionally, head activator attenuated tracheal tissue alterations in rats subjected to hypoxia [75]. Lebed’ko and colleagues proposed that normalisation of the respiratory epithelial and myocyte proliferation under head activator treatment may be used to modify the pathologic tissue remodelling observed in bronchopulmonary disorders such as asthma and chronic obstructive pulmonary disease [72]. These disorders are associated with epithelial abnormalities and hypertrophy of the smooth muscles of the bronchi [89]; head activator has displayed exactly the opposite effects, which could alleviate these anomalies [72].

The gastrointestinal system seems to also be affected by head activator. The peptide led to an increase in the proliferation of gastric epithelium [74] and secretion of amylase in cultured pancreatic cells [86]. Interestingly, the duodenal epithelium did not respond to head activator [85]. Under prenatal hypoxia, administered head activator normalised the rate of hepatocyte proliferation [75]. Furthermore, normalisation of the hepatic protein content and dose-dependent modulation of the hepatic ornithine decarboxylase, an enzyme that synthesises polyamines and reflects the hepatocyte metabolic and mitotic activity [82,83], was observed. These findings point to hepatoprotective properties, which may be interesting for the treatment of liver disorders.

There are also cardiovascular implications for head activator peptide. Fedoseev and colleagues demonstrated remodelling of the myocardial tissue upon compensatory left-ventricular hypertrophy induced by an artificial coarctation of the aorta treated with head activator [77,78]. The structural changes in the myocardium showed spatial and temporal dynamics; in particular, the histological alterations were most prominent in the subepicardial layer—the peptide influenced the cardiomyocyte size, extent of fibrosis, and capillary area [77,78], as well as increased proliferation in the rat myocardium [79]. These interesting findings demand further analysis of head activator effects in the scenario of heart failure and cardiomyopathies.

Moreover, head activator reduced the stress response in animals by regulating the hormonal and immune axes and attenuated lipid peroxidation. Head activator normalised glutathione peroxidase (GPO) activity and elevated α-tocopherol and total lipid levels [75,76]. Additionally, the peptide increased the thymocyte proliferation rate and number of peripheral blood lymphocytes compared to the normal references in rats, suggesting an immunomodulatory activity [80]. In rats subjected to stress by immobilisation, head activator normalised the thyrotropic-stimulating hormone (TSH), triiodthyronine (T3), thyroxine (T4), and corticosterone levels [74]. The peptide attenuated lipid peroxidation by reducing the lipoperoxide content in blood [74]. These combined studies reveal a cytoprotective and pro-regenerative potential of head activator.

Administration of head activator peptide to rats not subjected to any kind of stress revealed dose-dependent changes in hormone levels. At a lower dosage (10 μg/kg), β-endorphin was downregulated, while noradrenaline and corticosterone were upregulated; at a higher dosage (100 μg/kg) β-endorphin, T3 and insulin were upregulated, while corticosterone and noradrenalin were downregulated [81]. These hormonal alterations seem to follow a logical pattern, in the sense of a fight-or-flight reaction; the low-dose effect activates the stress-related catabolic hormones, while the high-dose effect activates the opposite steady-state anabolic activation.

The mechanism that underlies hormonal alterations induced by head activator has remained unclear. Kalenikova and colleagues showed that radiolabelled head activator is enriched in the pituitary gland and the pineal body after intravenous administration [72]. The pituitary gland, being a circumventricular organ, is supplied by fenestrated capillaries that allow for hormones to be secreted into the blood stream, in contrast to the mostly impenetrable blood–brain barrier in other brain regions [90]. The pituitary enrichment of head activator is likely attributed to the special characteristics of the blood–brain barrier in this region. We do not rule out that this might enable head activator to penetrate the barrier and regulate the secretion of pituitary hormones, thus resulting in the described hormonal disturbances. Our assumption also refers to the results of Drozd and colleagues, who discovered an antiemetic effect of head activator in a cat model [84]. Area postrema, the emesis centre located in the brainstem, is also a circumventricular organ, which contains an incomplete, leaky blood–brain barrier [91]. The antiemetic effect may be dependent on the modulation of this brain region.

The great body of scientific literature on head activator points towards a multifunctional nature. Due to its multiple effects and pleiotropic signalling, head activator peptide can be considered as an appealing therapeutic molecule.

Small bioactive molecules can trigger pleiotropic biological responses with high affinity and specificity, while tending to have low toxicity, immunogenicity, and production costs, compared to classic antibody-based biologicals [92]. The therapeutic peptides cover a wide range of applications, from simple hormone mimetics, such as insulin analogues, to more complex compounds, such as GLP-1 receptor agonists in diabetes and obesity therapy, an osteoanabolic fragment of parathormone teriparatide, somatostatin mimetics, anabolic ghrelin mimetics used to treat cachexia in cancer patients, and glatiramer acetate for multiple sclerosis [92]. However, there is a huge demand for therapeutic molecules to counteract nervous system disorders. Cnidarian peptides could set new frontiers in regeneration, particularly in the nervous system, due to their morphogenic nature, which enables cell proliferation and the de novo formation of new tissues.

## 6. Is Head Activator Evolutionarily Conserved?

The reproducible biological activity of synthetic head activator exogenously applied to mammalian systems raises the question of whether the peptide itself, or elements of its signalling cascade, are evolutionarily conserved. Of note, the molecular origin of head activator is still unclear. 

Originally, the peptide was found in *Hydra vulgaris* and also in *Anthopleura elegantissima* [28]. An identical peptide was subsequently detected in human, bovine and rodent hypothalamus, placenta, intestine, and blood [33,88,93,94,95], as well as in biopsies and serum samples from patients with neural and neuroendocrine tumours [96,97]. Outside of mammals, no evidence has been reported for the presence of head activator in other vertebrate species, to date. Head activator has been historically considered the only morphogenic peptide conserved between *Cnidaria* and *Mammalia*, since no other homologs could be detected in cnidarian and bilaterian neuropeptides and their receptors [17]. This distribution presents a notable phylogenetic discontinuity, as reports are limited to a small number of cnidarians and mammals. Furthermore, endogenous expression of head activator has been observed in P19 embryonal carcinoma cells [68], NH-15-CA2 neuroblastoma–glioma cells [44,69], NT2 neuronal precursor cell line [60], and in BON neuroendocrine cell line [60,70].

It should be emphasised that reported experiments on detection of head activator in both mammals and hydra have been carried out using chromatographic purification and sequencing or immunostaining approaches. These methods, especially immunocyto- and histochemical stainings, may not allow for confident confirmation of the peptide’s identity without additional control methods, such as immunoblotting, to rule out cross-reactivity with structurally similar epitopes. Several database entries in UniProt list head activator sequences for mammalian species; however, these records originate from those same biochemical isolation studies and are not derived from specific genomic studies. Despite extensive efforts, a gene-encoding head activator has not been conclusively identified. Schaller and colleagues attempted to identify the corresponding gene using molecular cloning approaches, without success. Even after comprehensive sequencing of hydra’s genome, no gene-encoding head activator precursor has been revealed, suggesting that the peptide is not derived from a canonical preprohormone gene. In addition, peptide screening within the Hydra Peptide Project has not identified a peptide corresponding to head activator. These findings have entailed a long-standing debate as to whether head activator is a purification artefact rather than an endogenous peptide [21]. Both possibilities are discussed in the review. It becomes evident that there is a notable paradox, in which a peptide with reproducible biological activity across multiple experimental system lacks clear genetic evidence for its endogenous origin.

Detecting homologs or orthologs in phylogenetically distant taxa, such as *Cnidaria* and *Mammalia*, is an untrivial task, since such extreme phylogenetic divergence may result in a loss of evolutionary information. Current evidence suggests that the peptidergic systems of *Cnidaria* and *Bilateria* evolved mostly independently from each other [17]. Despite this fact, we can still see parallels in the basic principles of cnidarian and bilaterian peptidergic signalling. The peptides are usually synthesised as preprohormones containing multiple copies of a peptide or even multiple different peptides that are liberated via proteolytic processing [17]. Some basic structural features are shared by *Cnidaria* and *Bilateria*, e.g., the N-terminal pyroglutamylation and the C-terminal amidation [17]. The neuropeptidergic signalling in *Cnidaria* and *Bilateria* is usually dependent on G-protein-coupled receptors (GPCRs); however, only the leucine-rich repeat-containing GPCRs have been identified to be conserved between *Cnidaria* and *Bilateria* [17].

Regardless of the strongly divergent sequences of orthologous proteins, it seems that the 3D structure of proteins can sometimes remain preserved between distantly related species. For example, tissue inhibitors of the matrix metalloproteases (TIMPs) are unified by a universal prototype structure that is significantly congruent, even in distant taxa [98]. It is conceivable that structural resemblances may allow for interactions of head activator with mammalian receptors, even in the absence of strong sequence preservation. Thus, the biological activity of synthetic head activator may reflect functional mimicry rather than direct evolutionary conservation.

Alternatively, if head activator is not genetically encoded, it may be generated by non-canonical mechanisms such as proteolysis or post-translational modifications. Additionally, the short half-life of head activator, estimated to be less than 30 seconds [72], may further hinder reliable detection. The question of the peptide’s evolutionary conservation remains unresolved. Regardless of its origin, however, the reproducible biological activity of synthetic head activator suggests that the peptide sequence may represent a bioactive motif with potential relevance for regeneration and neuroprotection.

## 7. Signalling Pathway of Head Activator Peptide in Mammals

**Figure 6:**
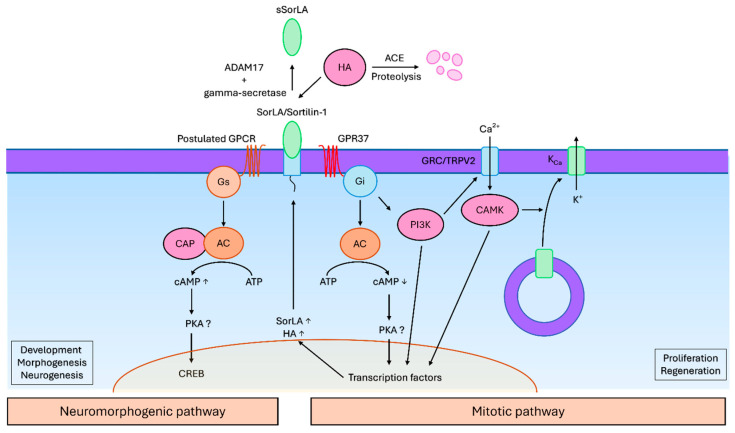
Signalling pathways of head activator. Head activator peptide context-dependently activates two functionally different signalling pathways—a mitotic and a neuromorphogenic cascade. The central receptor for both cascades is the SorLA transmembrane protein. It forms a complex with an inhibitory GPCR to initiate mitosis, or a stimulatory GPCR (potentially GPR37) to induce neuronal differentiation. In the neuromorphogenic pathway, a Gs protein activates the adenylyl cyclase (AC) to synthesise cyclic adenosine monophosphate (cAMP) as a second messenger, which in turn modulates the expression of target genes through the CREB transcription factor. In the mitotic pathway, a Gi protein is activated, leading to a depletion of the cAMP concentration. In parallel, the Gi protein activates the phosphoinositide 3 kinase (PI3K), which activates the GRC/TRPV2 calcium channel. The resulting calcium influx activates the calmodulin kinase (CAMK), which induces an externalisation of the calcium-dependent potassium channel (KCa) and consequently a calcium-dependent potassium efflux and hyperpolarisation. Following the ion rearrangements and kinase activations, mitosis occurs, likely accompanied by transcription factors regulating the necessary genes.

The signalling pathway of head activator in mammalian cells seems to occur in a similar manner compared to the cnidarian pathway (Figure 6). The first step is the binding of head activator to the VPS10 domain [64,99] of SorLA (SORL1, sortilin-related protein) [60]. This transmembrane receptor can exist in a membrane-bound form and in an extracellular soluble form generated by metalloprotease shedding [60], analogously to head activator-binding protein in the cnidarian pathway [61]. The proteolytic processing occurs through ADAM17 (disintegrin and metalloprotease 17, also known as TACE or tumour necrosis factor-α-converting enzyme) and gamma secretase [100]. Interestingly, a structurally and functionally related receptor sortilin-1, also has a low affinity to head activator [64]. It becomes thus clear that SorLA and sortilin-1 are involved in head activators’ mechanism of action. The unbound head activator is rapidly degraded through proteolytic processing by angiotensin-converting enzyme [95]. Blocking SorLA with antisense oligonucleotides has neutralised the mitogenic effect of head activator in NT2 cells, favouring its mitogenic signalling through SorLA [60]. The subsequent molecular response that controls mitosis occurs in a pertussis toxin-sensitive Gi-protein-dependent manner [69,70], in contrast to the cAMP response that controls neurogenesis, which would require a Gs protein [56]. This is consistent with the cnidarian pathway, where the cAMP axis is also being regulated, which then points to the likelihood of a forming SorLA/GPCR complex. Additionally, the previously described findings of Jacobsen and colleagues also support the receptor complex as a signalling mediator [64]. 

Rezgaoui and colleagues reported that the involved inhibitory GPCR is the orphan GPR37, which seems to exist in a complex with SorLA and internalises upon head activator treatment [65]. Furthermore, a direct ligand–receptor interaction between head activator and GPR37 was observed [65]. Another work has also detected clustering and internalisation of the GPR37 and inhibition of the adenylyl cyclase upon treatment with head activator [101]. Meyer and colleagues have noted that head activator and prosaptide (another putative ligand of the GPR37) have a similar C-terminal region, favouring head activator/GPR37 interaction [102]. Controversially, Dunham and colleagues could not reproduce such an interaction [103]. In an attempt to deorphanise some GPCRs, a high-throughput β-arrestin screening assay for potential ligands has also failed to show any association between head activator and GPR37 [104]. Another author has also expressed concerns regarding the interpretation of the results obtained by Rezgaoui and Gandía, since the presented images were not entirely consistent with the receptor internalisation [105].

After binding to its receptors, the head activator/SorLA complex (and possibly GPR37) becomes internalised [60,65]. In parallel, the expression of SorLA rises in a sense of an autocatalytic feedback loop and the unbound pool of SorLA in the intracellular compartment translocates to the cell surface [60].

This culminates in a calcium influx and calcium-dependent (gardos-type) potassium efflux, leading to a hyperpolarisation of the cells [69,70]. The initial calcium influx is provided by a transient receptor potential cation channel subfamily V member 2 (TRPV2), previously known as growth factor-regulated calcium channel (GRC), which externalises from the intracellular compartment to the cell surface [106]. This translocation requires the phosphoinositide-3-kinase and calmodulin-kinase [65,106]. The following steps of the cascade have not yet been characterised. A reasonable assumption is that the shifted ion levels and activated kinases might affect transcription factors, such as CREB, to regulate the expression of developmental and morphogenic genes.

The signalling cascade of head activator that controls neuronal differentiation in higher animals has not been extensively studied; however, there are indications that it may occur in a similar way to the previously described cnidarian cAMP–PKA–CREB pathway. While working on cultured embryonal chicken brain cells, Kajiwara and Sato have found that head activator peptide administration has led to a rise in cAMP, total protein concentrations, and the incorporation of radiolabelled nucleotides [71], as seen also in hydra. The studied culture was chosen to be a mixture of all cell types present in the brain; due to technical limitations, a distinction between neuronal and glial or precursor cell response was not possible. The incorporation of the nucleotides has been attributed to the proliferation of dividing cells; the rising cAMP concentration has been related to the development of post-mitotic differentiating cells [71]. Similarly, in injured hepatic tissue from rats, head activator was shown to induce a biphasic response, with the initially elevated cAMP concentration attributed to the inhibition of the repair processes, and later on, with cAMP reduction related to the activation of tissue repair [57]. Both phases were accompanied by inversely oriented cyclic guanosine monophosphate (cGMP) concentration changes [57]. This corroborates the hypothesis that head activator shifts the GPCR activity between inhibitory and stimulatory G-proteins, and, thus, temporally changes its mode of action, dependent on the differentiation status of the target cells, similar to the cnidarian pathway.

Whether head activator can act as a ligand for the GPR37 receptor has been a subject of heated discussion in the scientific community. This conflict is likely the reason why research on head activator was not pursued further. Experiments on GPR37 are methodically a challenging undertaking, since its expression is mainly restricted to the cytosol in heterologously overexpressing cells; few receptor molecules are exposed on the cell surface, leading to no apparent activation or ligand binding [107]. Southern and colleagues did not address this factor. To overcome this difficulty Dunham and colleagues have ensured that their construct had sufficient surface presentation of GPR37 through several different approaches: N-terminal truncation of the receptor or its co-expression with syntenin-1 [103]. This approach, however, did not yield any head activator-induced response, presenting strong evidence against head activator/GPR37 interaction. However, we must mention that neither Dunham nor Southern have tested their cell constructs for the expression of SorLA, the putative component of head activator receptor complex; hence, one component of the signalling cascade could have been missing. This could mean that GPR37 alone is not enough to induce a response and may only signal in complex with SorLA. Another point of criticism around Dunham’s work is the absence of any control condition to compare with the head activator treatment. It is unclear whether GPR37 truncation or syntenin-1 co-expression, performed by Dunham and colleagues, could completely abolish the receptor activity, and that, therefore, no head activator-induced response has been observed. An alternative way to increase receptor surface recruitment without potentially interfering with the receptor activity could be the treatment with chemical chaperones such as 4-PBA [108]. These results need to be reviewed with caution. We believe that more appropriate (cell) models need to be established to unravel the functions of head activator. In particular, considering the primarily neuronal origin and activity of head activator, the choice of neuronal cells/cell lines seems to be more appropriate; furthermore, the membrane localisation of the GPR37 receptor and its co-expression with SorLA must be ensured. Confirmation studies using primary neuronal cells and/or their precursors cells rather than immortalised cell lines as well as in vivo testing would help to find an answer. Altogether, according to the available literature, there is stronger evidence for SorLA than GPR37 as a component of head activators’ signalling pathway; the GPR37 involvement has yet to be confirmed, but it should not be dismissed. We believe that a broader approach, without focusing on a single receptor, would be a fitting way to study head activator signalling. For instance, qPCR analysis of a broad receptor panel or a mass spectrometric analysis of a pull-down fraction against an immobilised head activator could shed more light on the specific signalling cascade.

## 8. Potential Role of Head Activator in Regenerative Medicine and Human Neuropathology

As discussed in previous sections, synthetic head activator exerts pleiotropic effects in multiple mammalian tissues and cell types and can induce systemic hormonal changes. Reported targets include epithelial cells, smooth myocytes, cardiomyocytes, thymocytes, hepatocytes, endocrine axes, such as the thyroid, catecholamine and insulin homeostasis, as well as components of the lipid peroxidation system in vivo. In addition, neurotropic and proliferative effects have been observed in multiple human cell lines. These observations suggest a potential use of head activator as a modulator of regenerative processes by inducing proliferation and repair in injured tissues, thereby enabling targeting of multiple organs and systems. Further relevance in the context of neurological disorders, where stimulation of neuronal survival, differentiation, or repair processes represent an important therapeutic goal, is given by considering the neuromorphogenic roles of head activator. Both detected receptors for head activator, SorLA and GPR37, show interesting pathophysiologic connections to several neuropathologic conditions, and may, therefore, represent potential drug targets. 

SorLA has been reported to participate in the pathogenesis of Parkinson’s [109] and Alzheimer’s disease, atherosclerosis, type 2 diabetes, obesity [63], HER-2 driven cancers, such as breast and ovarian cancer [110,111,112], and in immune-mediated processes such as neuroinflammation and pathogen defence [113]. SorLA belongs to the group of VPS10 domain receptors and is mostly associated with the Golgi apparatus, where it regulates the intracellular trafficking of various proteins [63]. The receptor molecule is structurally similar to low-density lipoprotein receptor and can also internalise lipoproteins into cells through interaction with Apolipoprotein E [63]. Furthermore, SorLA can bind and facilitate intracellular transport of glial-derived neurotrophic factor and its receptors [114]. Under inflammatory conditions, SorLA regulates cytokine secretion, and, thus, the activity of microglia, macrophages, and lymphocytes, as observed in neuroinflammatory, neurodegenerative, and even psychiatric disorders [113]. Truncated variants of SorLA are associated with the development of Alzheimer’s disease [115]. Wild-type SorLA is a protective factor that binds amyloid precursor protein and prevents its proteolytic processing into peptides that can nucleate into cytotoxic aggregates [116,117,118]. Parkinson’s disease is a major pathology that is associated with GPR37 abnormalities [119]. GPR37, also known as rhodopsin-like orphan G-protein-coupled receptor 37 or parkin-associated endothelin receptor-like receptor, is hypothesised to act as a neuropeptide receptor [119,120]. Its ligand is currently unknown, and head activator has not been recognised as such. GPR37 is expressed in neurons, microglia and oligodendrocytes and coupled to a Gi/o protein that signals through the MAPK/Erk pathway [119]. In Parkinson’s disease, the misfolded GPR37 receptor forms cytotoxic aggregates responsible for the degeneration of the dopaminergic neurons. GPR37 fragments in the cerebrospinal fluid were suggested to be used as a biomarker of the disease’s progression—interestingly, different fragment patterns of the receptor were detected in the idiopathic and atypic variants of Parkinson’s disease, allowing for differentiation between these phenotypes [121]. Under normal conditions, the native GPR37 has a neuroprotective role against oxidative stress and ischemia [119,122]. GPR37 has been linked to epilepsy, autism, neuroinflammation, and demyelination [119]. Loss of GPR37 has been associated with premature hypermyelination [123] and a higher likelihood of demyelination in a cuprizone model of multiple sclerosis [124], while preventing neurodegeneration in Parkinson’s disease models [125]. Apart from the nervous system, GPR37 is involved also in spermatogenesis and carcinogenesis [119].

With respect to head activator, it is important to note that endogenously expressed head activator has been found in immortalised brain tumour cell lines [34,69] and in the human material of meningeomas, astrocytomas, and glioblastomas [96]. The patients also displayed elevated systemic levels of head activator in their blood, which normalised after the tumour resection, thus highlighting head activator as a potential neurooncological biomarker [96,97]. If head activator is, indeed, endogenously expressed and supports neurogenesis, it is reasonable to assume that different growth factors, including the molecule itself, might be expected to be elevated in tumorous brain tissue. However, one study found reduced levels of head activator in astrocytoma in comparison to the normal tissue [126]. To explain these discrepancies, Ekman and colleagues postulated the different specificity of the antibodies used in both studies. Another important fact to note is that head activator is exclusively a biomarker for neuronal and not glial tumours, as this neuropeptide is produced and stored in neurosecretory granula. In addition, we must also consider the high heterogeneity of brain tumours, where the expression of head activator could vary significantly depending on type, characteristics, and grading.

**Figure 7:**
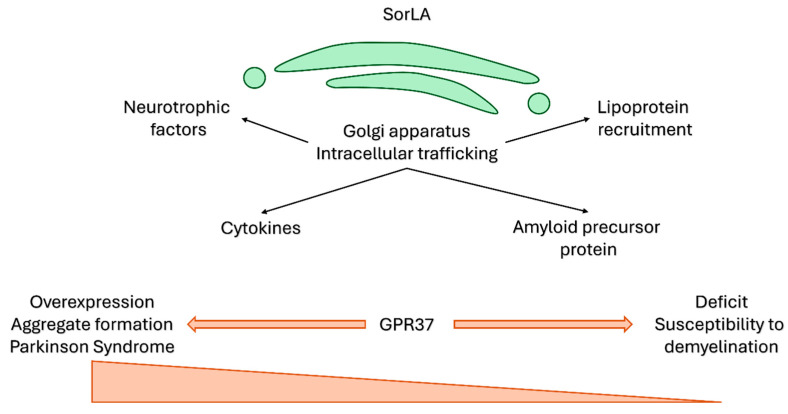
SorLA and GPR37 in human neuropathology. Two major putative receptors involved in head activator signalling are the SorLA and GPR37. SorLA is mostly an intracellular protein of the Golgi apparatus, with pleiotropic functions ranging from the intracellular transport of neurotrophic factors and cytokines to a lipoprotein receptor-like ability to bind and internalise lipoproteins and amyloid precursor protein processing. This rich functional variability is also mirrored in the pathogenetic involvement of SorLA in multiple disorders. Similarly, GPR37 homeostasis was also shown to participate in pathogenesis of neurological disorders. Its overexpression results in the formation of neurotoxic aggregates, observed in Parkinson’s disease; likewise, GPR37 deficiency is also pathological, and can lead to demyelination.

In summary, it seems that head activator’s signalling pathway may enable engagement with different pathologic conditions, particularly those of the nervous system. SorLA and GPR37 are interesting targets that place head activator at the core of many neurological diseases (Figure 7).

Addressing head activator and its putative receptors may provide an opportunity to obtain useful insights into neuropathology and maybe identify novel therapeutic drugs. Alone, the interaction of head activator with SorLA is a significant framework applicable to neurological disorders. Furthermore, the association of head activator with GPR37 is valuable for the neuropathology field.

## 9. Conclusions

In this work, we focused on one cnidarian morphogen, head activator, to revisit and discuss its roles in neurogenesis and regeneration. In hydra, head activator regulates regeneration and development of the head region, and, concurrently, nervous net maintenance, by determining the neuronal fate of interstitial cells. Intriguingly, this peptide is active in both cnidarian and mammal tissues with similar effects. Multiple preclinical studies using rodents could demonstrate the mitogenic effects of exogenously administered head activator across various organs. In addition to mitosis, the peptide can also influence myocardial structure, hormonal axis, and lipid and liver metabolism. Indirectly, cell culture experiments on various neuronal cells could assess the neurogenic activity of head activator, as observed in hydra. All these findings strongly suggest that head activator could be a potent multifunctional molecule. Despite such compelling evidence, head activator has been largely forgotten since the last publication on head activator in the early 2000s. The reason for this is likely due to several controversial findings on its evolution and signalling. First, the gene of origin could never be identified; some findings reporting on head activator’s detection in mammalian tissues could not be reproduced. Furthermore, one of head activator’s putative receptors, the orphan GPR37, has been heavily disputed. The question of head activator’s evolutionary conservation requires further experimental testing to give a definitive answer bridging the primordial nervous nets of cnidarians and the complex nervous systems of mammals. The experimental evidence for the peptide to act as an exogenous ligand on mammalian systems is intriguing. We have summarised reported findings on the signalling cascades controlled by head activator: the peptide employs at least two different context-dependent pathways that are activated based on the mitotic activity and differentiation status of the targeted cells. The central receptor of head activator seems to be the SorLA protein, which most likely engages in a complex with both stimulatory and inhibitory G proteins to facilitate further signalling through regulation of the cAMP axis. We have also collected and discussed in-depth critical aspects around the head activator/GPR37 interaction. We also hypothesise that head activator may be a central link between multiple neuropathologies ranging from neurodegeneration to myelination disorders including Alzheimer’s and Parkinson’s diseases, as well as multiple sclerosis.

## Figures and Tables

**Table 1:** Morphogenic and neurogenic cnidarian peptides.

Peptide	Effect	Reference
**morphogenesis**		
pedibin (Hym-346)	foot formation and regeneration head patterning	[29]
HEADY	head formation budding	[31]
**neurogenesis**		
Hym-355	stem cell proliferation stimulation of neuronal differentiation	[32]
Hym-33H	inhibition of neuronal differentiation	[24]
Hym-35		
Hym-37		
Hym-310		
**morphogenesis and neurogenesis**		
head activator	head formation and regeneration stem cell proliferation stimulation of neuronal differentiation	[33]
pedin (Hym-323)	axial body patterning foot formation and regeneration budding stem cell proliferation stimulation of neuronal differentiation	[29]

**Table 2:** Context-dependent modes of action of head activator peptide.

Condition		Effect
cell type	mitotic	mitosis and differentiation
	post-mitotic	differentiation
time point	early	mitosis
	late	differentiation
concentration (receptor affinity)	low (high)	mitosis
	high (low)	differentiation
pathway (AC-cAMP-PKA axis)	AC inhibition	mitosis
	AC activation	differentiation, neurogenesis

**Table 3:** Effects of head activator in mammals.

Tissue/Cell Type/Hormone	Effect	Reference
rat corneal epithelium	proliferation ↑	[73,74]
rat lingual epithelium	proliferation ↑	
rat tracheal epithelium	proliferation ↑	[75,76]
rat tracheal smooth muscle cells	proliferation ↓	
lipid peroxidation system in hypoxic rats	inhibition in lungs	
stress response in rats	reduction of lipid peroxidation, corticosterone, normalised thyrotropic hormone, T3, T4, and thymus weight	[74]
rat myocardium upon a left ventricular hypertrophy	myocardial remodelling	[77,78]
rat myocardium	proliferation ↑	[79]
rats exposed to prenatal hypoxia	normalisation of thymocyte proliferation	[80]
rats exposed to prenatal hypoxia	normalisation of hepatocyte and tracheal epithelium proliferation, reduction of pulmonary lipid peroxidation	[75]
rat β-endorphine	low dose: ↓ high dose: ↑	[81]
rat corticosterone	low dose: ↑ high dose: ↓	[81,82,83]
rat noradrenaline	low dose: ↑ high dose: ↓	
rat thyroid hormones	low dose: = high dose: Τ3 ↑	
rat insulin	low dose: = high dose: ↑	
liver function in partially hepatectomised rats	ornithine decarboxylase activity in low/medium/high dose: =/↑/↓ protein content: ↑	
cat model of motion sickness	antiemesis	[84]
rat duodenal smooth myocytes	proliferation =	[85]
injured rat liver	4 h after injury: cAMP ↑, cGMP = 24 h after injury: cAMP ↓, cGMP ↑	[57]
injured rat striated muscle	cAMP =, cGMP =	
rat cultured pancreatic cells	amylase secretion ↑	[86]
human erythrocytes	activation of Na/H exchange	[87]

↑: upregulation, ↓: downregulation, =: unchanged.

## Data Availability

Not applicable.
